# Multifold Fermions and Fermi Arcs Boosted Catalysis in Nanoporous Electride 12CaO·7Al_2_O_3_

**DOI:** 10.1002/advs.202205940

**Published:** 2022-12-27

**Authors:** Weizhen Meng, Xiaoming Zhang, Ying Liu, Xuefang Dai, Guodong Liu, Yuantong Gu, E. P. Kenny, Liangzhi Kou

**Affiliations:** ^1^ State Key Laboratory of Reliability and Intelligence of Electrical Equipment Hebei University of Technology Tianjin 300130 China; ^2^ School of Materials Science and Engineering Hebei University of Technology Tianjin 300130 China; ^3^ School of Mechanical Medical and Process Engineering Queensland University of Technology Garden Point Campus Brisbane QLD 4001 Australia

**Keywords:** multifold fermions, topological electrides, topological quantum catalysts

## Abstract

Topological materials have been recently regarded as ideal catalysts for heterogeneous reactions due to their surface metallic states and high carrier mobility. However, the underlying relationship between their catalytic performance and topological states is under debate. It has been discovered that the electride 12CaO·7Al_2_O_3_ (C12A7:4e^−^) hosts multifold fermions and Fermi arcs on the (001) surface near the Fermi level due to the interstitial electrons. Through the comparison of catalytic performance under different doping and strain conditions, based on the hydrogen evolution process, it has been demonstrated that the excellent catalytic performance indeed originates from topological properties. A linear relationship between the length of Fermi arcs, and Gibbs free energy (ΔG_H*_) has been found, which not only provides the direct evidence to link the enhanced catalytic performance and surface Fermi arc states, but also fully clarifies the fundamental mechanism in topological catalysis.

## Graphical abstract

This work discovers electride 12CaO·7Al_2_O_3_ possesses multifold fermions and long Fermi arcs originating from excess electrons in the interstitial sites. These topological components are fully demonstrated as the origin for catalytic enhancement in 12CaO·7Al_2_O_3_‐based catalysts. A quantitative relationship between a topological parameter and a catalytic parameter has been built for the first time.

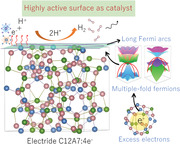

## 1 Introduction

Topological materials^[^ ^1^, ^2^, ^3^, ^4^, ^5^ ^]^ have been recently identified as prime candidates for catalysts in heterogeneous reactions because of their highly conductive and robust surface states. Topological semimetals were proposed as potential catalysts for the hydrogen evolution reaction (HER), including high‐topological‐charge semimetals in PtAl, PtSn_4_, Nb_2_S_2_C, and V_0.75_Ni_0.25_Al_3_, and nodal‐line semimetals in the TiSi family.^[^ ^6^, ^7^, ^8^, ^9^, ^10^ ^]^ Weyl semimetals of the TaAs family also demonstrated enhanced catalytic activity for HER.^[^ ^11^ ^]^ In addition, high catalytic performance in HER and CO oxidation have been reported in topological insulators Bi_2_Se_3_ and Bi_2_Te_3_.^[^ ^12^, ^13^, ^14^, ^15^ ^]^ These findings have indicated the feasibility of developing high‐performance catalysts from topological materials. However, the fundamental mechanism and the underlying relationship between catalytic performance and topological states are still unclear.

Electrides are promising candidates for investigating this question.^[^ ^16^, ^17^, ^18^, ^19^ ^]^ Owing to loosely‐bound excess electrons trapped in interstitial regions of the crystal lattice,^[^ ^20^, ^21^ ^]^ electrides usually exhibit high carrier mobility and low work function. This has been long believed as the reason for high catalytic activity in electride‐based catalysts. However, different mechanisms were proposed in other works. It was found that catalytic enhancement can be preserved even after the electride Ca_2_N is completely transformed into the Ca_2_NH hydride, where interstitial electrons are annihilated.^[^ ^22^ ^]^ Another work^[^ ^23^ ^]^ on the catalyst Ru/C12A7:4e^−^ (C12A7:4e^−^ represents 12CaO·7Al_2_O_3_) indicates that surface‐adsorbed hydrogen, rather than the hydride captured in the cages of electride C12A7:4e^−^, is responsible for the high catalytic activity of reactive hydrogen in ammonia synthesis. Conflicting explanations lead to confusions about the underlying mechanism — a deeper understanding is urgently required.

Some electrides possess intrinsic, nontrivial band topology.^[^ ^24^, ^25^, ^26^, ^27^, ^28^, ^29^, ^30^, ^31^, ^32^ ^]^ In particular, floating surface bands,^[^ ^26^ ^]^ not present in traditional topological materials, may have significant effects on catalysis. Inspired by these works, we have demonstrated C12A7:4e^−^ as a new topological electride with multifold fermions by way of theoretical calculations and modeling analysis. The multifold fermions originate from excess electrons, and are characterized with extremely long Fermi arcs. Notably, a linear relationship between catalytic enhancement and Fermi arc length has been established, providing direct evidence that the catalytic enhancement is indeed from the surface Fermi arc states. These findings have indicated the feasibility of developing high‐performance catalysts from topological materials. The mechanism for the catalytic enhancement in topological catalysts has been qualitatively attributed as the large density of surface states.^[^ ^13^ ^]^ However, an underlying quantitative relationship between catalytic performance and topological states have been obtained till now.

## 2 Results and Discussion

### 2.1 Topological Electride Properties

**Figure 1:**
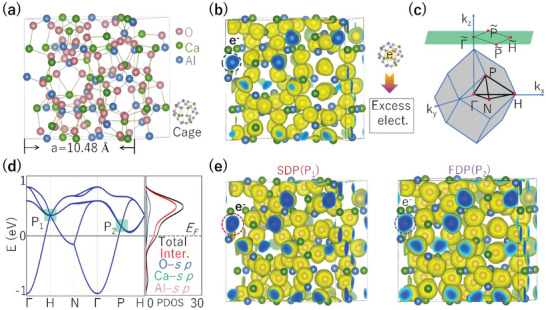
a) Crystal structure of electride C12A7:4e^−^. The cage structure is shown in the lower corner. b) The ELF of electride C12A7:4e^−^ with the isosurface values set as 0.65. The lower corner of (b) shows the confined electrons inside the cage. c) Bulk and (001) surface Brillouin zone of electride C12A7:4e^−^ with high symmetry points indicated. d) Band structure and PDOSs of electride C12A7:4e^−^. The band crossings at the H and P points are denoted as P_1_ and P_2_. “Inter.” denotes the interstitial electrons. e) The PED in the energy region of ±0.02 eV around P_1_ and P_2_. The isosurface value is chosen as 0.005 Bohr^−3^.

We start by characterizing the crystal structure and electride nature of C12A7:4e^−^ based on density functional theory (DFT) in Vienna ab‐initio simulation package (VASP) packages. As shown in **Figure** **1**a, it has a cubic structure with the space group of *I* $\bar{4}$ *3d* (No. 220). The unit cell contains 12 large cages, each ≈0.4 nm in diameter, depicted in Figure 1a. The DFT‐optimized structural parameters agree well with the experimental values,^[^ ^34^ ^]^ indicating the validity of our DFT methods. As shown by the electron localization function (ELF) in Figure 1b, there is a large region with confined electrons in the center of the cages (the interstitial 12b Wyckoff site, see the circled region). This result confirms that C12A7:4e^−^ is a typical electride. Considering the valence states in C12A7:4e^−^, it contains four excess electrons per unit cell, and each cage has the con‐centration fraction of 1/3 of an electron.

The electronic structure and partial density of states (PDOS) of C12A7:4e^−^ are shown in Figure 1d. C12A7:4e^−^ has two band crossing points located at the high symmetry points H and P near the Fermi level, as denoted by P_1_ and P_2_, respectively. From the PDOS, we find the bands within the energy window are mostly composed of interstitial electrons, which are confirmed from the partial electron density (PED) and the orbital component analysis in the energy region of ± 0.02 eV around *P*_1_ and *P*_2_ (see Figure 1e). The origin of the bands at the crossing points is also verified by the elementary band representations (BRs) based on topological quantum chemistry theory.^[^ ^35^, ^36^, ^37^ ^]^ These results show that the bands at *P*_1_ and *P*_2_ are floating bands with BRs of *A@12b*, from the interstitial electrons. Remarkably, the “BRs” represents the atomic orbital characteristics in real space and the band representation in momentum space. For electride C12A7:4e^−^, *A@12b* represents irreducible representations (*A*) of bands near Fermi level and Wyckoff sites (12b) of excess electrons. (As described in Table SI, Supporting Information^[^ ^33^ ^]^). These findings strongly support the ELF results in Figure 1b.

### 2.2 Effective Models

Based on symmetry analysis, we find *P*_1_ is a sixfold degenerate point (SDP), and *P*_2_ is fourfold (FDP). Specifically, P_1_ is located at the H point, and the point group at H point belongs to *T_d_*, which can generate four symmetry‐operations: $\left\{S_{4x}^{+}|\frac{1}{2}00\right\},\;\left\{M_{110}|\frac{1}{2}00\right\},\;\left\{C_{3,1\bar{1}\bar{1}}^{-}|1\frac{1}{2}\frac{1}{2}\right\}$ and time‐reversal symmetry (*T*). Furthermore, due to the six‐dimensional irreducible representation (*IR*) {*Г_6_, Г_7_*}, Six‐bands at H point are degenerated and are protected by the above four symmetry‐operations. (As described in Table SII and Figure S1, Supporting Information^[^ ^33^ ^]^). Under the basis constructed by these two IRs, the generating elements can be written as matrix representation forms, denoted as O(*R*), with *R* as the actions in momentum space. To be specific, the generating elements take the following under such a six‐dimensional IR,

(1) $$\begin{array}{cc} O\;\left(S_{4x}^{+}\right)=\sigma_{z}\;⊗\left(iW\right), & O\;\left(M_{110}\right)=\sigma_{z}⊗\left(iX\right),\;\\ O\;\left(C_{3,1\bar{1}\bar{1}}^{-}\right)=\sigma_{0}⊗A, & O\;\left(C_{3,1\bar{1}\bar{1}}^{-}\right)=\sigma_{x}⊗I_{3\times 3} \end{array}$$

Here, *I*_3×3_, and *σ*_0_ are the identity matrix, *σ*_*i*_ (i = *x*, *y*, *z*) is the Pauli matrix. And W, X, A are 3 × 3 matrixes, taking in the following forms,

(2) $$W\;=\left(\begin{array}{ccc} 0 & \;1 & \;0\\ -1 & \;0 & \;0\\ 0 & \;0 & \;-1 \end{array}\right),\;X=\left(\begin{array}{ccc} 1 & \;0 & \;0\\ 0 & \;0 & \;1\\ 0 & \;1 & \;0 \end{array}\right),\;A\;=\left(\begin{array}{ccc} 0 & \;0 & \;1\\ 1 & \;0 & \;0\\ 0 & \;1 & \;0 \end{array}\right)\;$$

Since the effective Hamiltonian at H point should respect the above symmetry constrains, such that the effective Hamiltonian should satisfy the following algebra relationship,

(3) $$O\left(R\right)H\left(Rk\right)O\;{\left(R\right)}^{-1}=\;H\left(k\right)$$

at H point. We consider up to the first order of ***k***. Consequently, the effective Hamiltonian can be written as

(4) $$H_{SDP}\;\left(k\right)=\left(\begin{array}{cc} h_{11}\left(k\right) & \;h_{12}\left(k\right)\\ h_{21}\left(k\right) & \;h_{22}\left(k\right) \end{array}\right)$$

Here, *h_ij_* (***k***) is a 3 × 3 matrix. Specifically,

(5) $$\begin{array}{c} h_{11}=\;-\;h_{22}=\;\gamma \left(\begin{array}{ccc} 0 & \;-k_{x} & \;k_{y}\\ -k_{x} & \;0 & \;k_{z}\\ k_{y} & \;k_{z} & \;0 \end{array}\right),\;\\ h_{12}=h_{21}^{†}\;=\;-\lambda \left(\begin{array}{ccc} 0 & \;k_{x} & \;k_{y}\\ -k_{x} & \;0 & \;-k_{z}\\ -k_{y} & \;k_{z} & \;0 \end{array}\right)\; \end{array}$$

with *λ* is a real parameter, and *λ* is a complex parameter. In fact, we can rewrite the effective Hamiltonian based on a 3 × 3 matrix, which is given as

(6) $$H_{S-1}\;\left(k^{\prime }\right)=\left(\begin{array}{ccc} 0 & \;-ik_{x}^{\prime } & \;ik_{y}^{\prime }\\ ik_{x}^{\prime } & \;0 & \;ik_{z}\\ -ik_{y}^{\prime } & \;-ik_{z} & \;0 \end{array}\right)\;=k^{\prime }\;\cdot S$$

with **k**′ = (*k_x_*,−*k_y_*,−*k_z_*). Since both ***k*** and ***k*** ′ are measured from the sixfold degenerate point at H point, there exists a coordinate transformation between them, in the following text, for simplicity, we neglect “′”. Here, ***S*** is the spin‐1 matrix, specifically,

(7) $$S_{x}=\left(\begin{array}{ccc} 0 & \;-i & \;0\\ i & \;0 & \;0\\ 0 & \;0 & \;0 \end{array}\right),\;S_{y}=\left(\begin{array}{ccc} 0 & \;0 & \;i\\ 0 & \;0 & \;0\\ -i & \;0 & \;0 \end{array}\right),\;S_{z}=\left(\begin{array}{ccc} 0 & \;0 & \;0\\ 0 & \;0 & \;i\\ 0 & \;-i & \;0 \end{array}\right)$$

satisfying the algebra of angular momentum [*S_i_*,*S_j_*] = *iε*_*ijk*_ *S_k_*. Therefore, the SDP at H point can be written as

(8) $$H_{\mathrm{SDF}}\;\left(k\right)=\left(\begin{array}{cc} -\gamma \;h_{11}\left(k\right) & \;-i\lambda H_{S-1}\left(k\right)\\ i\lambda H_{S-1}^{†}\left(k\right) & \;\gamma h_{22}\left(k\right) \end{array}\right)$$

Such that, in our case, when we consider a limitation that *γ* ≪ |*λ*|, the effective Hamiltonian can be regarded as a composition of two spin‐1 excitations of opposite chirality.

Similarly, there exist four independent symmetry‐operations at *P* point, namely $\left\{S_{4x}^{+}|\frac{1}{2}00\right\},\;\left\{C_{3,1\bar{1}\bar{1}}^{+}|1\frac{1}{2}\frac{1}{2}\right\},\;\left\{C_{2y}|0\frac{1}{2}\frac{1}{2}\right\},\;\left\{C_{2x}|\frac{3}{2}\frac{3}{2}0\right\}$. Due to the existence of four‐dimensional IR {*Г*_4_}, a fourfold degenerate point are formed at P point and protected by the above four symmetry‐operations. (As described in Tables SII and Figure S1 of Supporting Information^[^ ^33^ ^]^). We can also write the effective Hamiltonian in this IR. Before proceeding, we consider the matrix representations of symmetry operations, they can be given as,

(9) $$\begin{array}{c} O\;\left(C_{32}^{+}\right)=\left(\begin{array}{c} \begin{array}{cc} \begin{array}{cc} \frac{\sqrt{2}}{4}e^{i\pi /4}, & \;\frac{\sqrt{2}}{4}e^{-i\pi /4},\\ \frac{\sqrt{2}}{4}e^{i\pi /4}, & \;-\frac{\sqrt{2}}{4}e^{-i\pi /4}, \end{array} & \;\begin{array}{cc} \frac{\sqrt{6}}{4}, & \;-i\frac{\sqrt{6}}{4}\\ \frac{\sqrt{6}}{4}, & \;i\frac{\sqrt{6}}{4} \end{array} \end{array}\\ \begin{array}{cc} \begin{array}{cc} -i\frac{\sqrt{6}}{4}, & \;-\frac{\sqrt{6}}{4} \end{array}, & \;\begin{array}{cc} \frac{\sqrt{2}}{4}e^{i\pi /4}, & \;\;\frac{\sqrt{2}}{4}e^{-i\pi /4} \end{array}\\ \begin{array}{cc} -i\frac{\sqrt{6}}{4}, & \;\frac{\sqrt{6}}{4} \end{array}, & \;\begin{array}{cc} \frac{\sqrt{2}}{4}e^{i\pi /4}, & \;-\frac{\sqrt{2}}{4}e^{-i\pi /4} \end{array} \end{array} \end{array}\right)\\ O\;\left(C_{2y}\right)=\sigma_{0}⊗\sigma_{x},\;O\;\left(C_{2x}\right)=-\sigma_{0}\;⊗\sigma_{z},\\ \;O\;\left(S_{4x}\right)=\frac{\sqrt{2}}{2}\;e^{i\pi /4}\left(\sigma_{z}⊗\sigma_{0}-i\sigma_{z}⊗\sigma_{0}\right) \end{array}$$

Since the effective Hamiltonian at P point follows these symmetry operations, such that it can be given by,

(10) $$H_{FDP}\;\left(k\right)=\left(\begin{array}{cc} g_{11}\left(k\right) & \;g_{12}\left(k\right)\\ g_{21}\left(k\right) & \;g_{22}\left(k\right) \end{array}\right)\;$$

Among them,

(11) $$\begin{array}{c} g_{11\;}\;\left(k\right)=\;-g_{22}\;\left(k\right)=\;v\left(k_{-}\sigma_{+}+h.c.\right),\\ g_{12\;}\;\left(k\right)=g_{21}^{*}\;\;\left(k\right)=\;-c_{1}k_{z}\sigma_{z}+c_{2}\left(k_{+}\sigma_{+}+h.c.\right) \end{array}$$

Here, *v* is a real parameter, *c*′s are complex parameters. To be specific, $c_{1}=\;Ae^{i\theta_{1}}$, $c_{2}=\;Be^{-i\theta_{2}}$, and A and B are real parameters. In fact, there exist only two independent parameters for this Hamiltonian, we denote them as *α* and *β*. And, *v* = 3*α* + *β*, *A* = |3*α* − *i*(*α* + 4 *β*)|, *B* = |3*α* + *i*(2*α* + *β*)|, *θ*_1_ = *arg*|3*α* − *i*(*α* + 4 *β*)|, *θ*_2_ = *arg*|3 *β* + *i*(2*α* + *β*)|.

### 2.3 Fermi Arc Surface States

**Figure 2:**
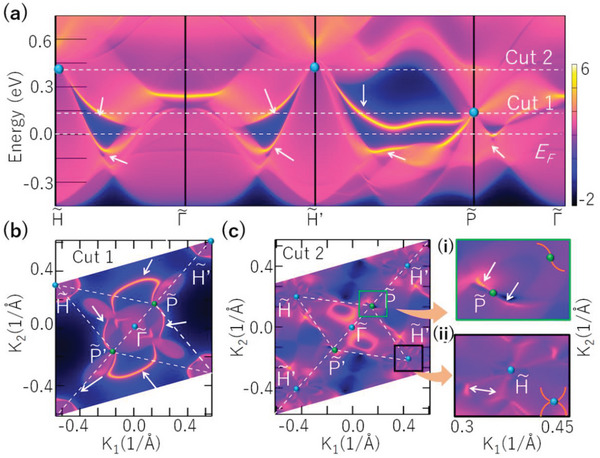
a) The (001) surface states of electride C12A7:4e^−^. b,c) show the (001) surface slice undercut 1 and cut 2 (the energy of cut 1 and cut 2 are taken exactly at SDP and FDP), respectively. In (a), (b), (c), the Fermi arcs are indicated by the white arrows. In (c), the illustrations on the right panel are the partially enlarged view of the states in the black (i) and green (ii) boxes.

The above analysis clearly identifies C12A7:4e^−^ as a topological electride with the coexistence of SDP and FDP fermions. Usually, the SDP and FDP are expected to exhibit surface Fermi arc states.^[^ ^37^ ^]^ **Figure** **2**a shows the (001) surface states along the paths shown in Figure 1c. Two sections of Fermi arcs originating from the $\tilde{H}$ ($\tilde{H^{\prime }}$) and $\tilde{P}$ points are observed. As shown in Figures 2b and c, these Fermi arcs can also be observed by the surface slices near the Fermi level (cut 1 at 0.146 eV and cut 2 at the 0.346 eV). The surface slices show that the arcs of SDP exhibit a dumbbell‐like shape and cover a large region of the (001) surface (see Figure 2a,c). In contrast, two sections of FDP Fermi arcs have a petal‐like shape (see Figure 2a,b), which are extremely long, nearly traversing the entire surface of the Brillouin zone.

**Figure 3:**
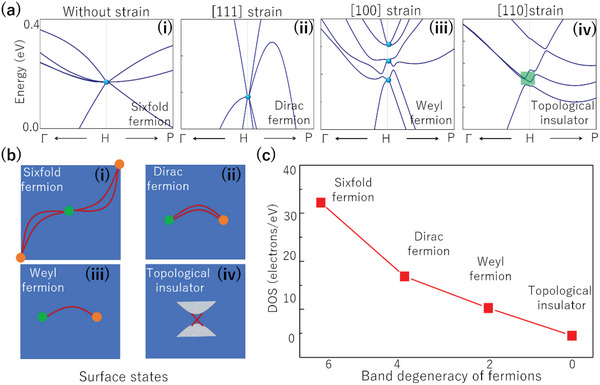
a) The electronic band structures along *k*‐paths Г‐H‐P under different strains (namely without strain (i), and 0.5% strain along [111], [100], [110] directions (ii‐iv), respectively) for electride C12A7:4e^−^. These crossing points show six‐, four‐(Dirac), two‐fold (Weyl) and topological insulator, respectively. b) The surface states (i‐iv) corresponding to six‐, Dirac, Weyl fermions and topological insulator, where the red lines represent the Fermi arcs (six‐, Dirac‐, Weyl‐fermions) and Dirac cone (Topological insulator) surface states. c) The quantitative relationship between band degeneracy of different fermions and DOSs near the Fermi level on the (001) surface.

Similar to traditional Weyl/Dirac topological quantum catalysts such as TaAs and PtAl,^[^ ^6^, ^11^ ^]^ C12A7:4e^−^ naturally possesses topological bulk band structure and surface Fermi arc states. However, compared with these examples, C12A7:4e^−^ is different in two aspects: (1) C12A7:4e^−^ is an electride, and the multifold fermions are comprised of the excess interstitial electrons, (2) it possesses multifold fermions (SDP and FDP), with significantly longer Fermi arcs than their Weyl/Dirac counterparts. The SDP fermions in C12A7:4e^−^ can be transferred into Dirac/Weyl fermions and topological insulator by applying a certain strain (See **Figure** **3**a). These states correspond to different surface states (see Figure 3b). With the reduction of the band degeneracy of the fermions, we find the surface DOSs reduce (see Figure 3c). The length of Fermi arcs in SDP fermions is 1.682 Å^−1^, while the values are 0.164 Å^−1^ and 0.128 Å^−1^ for Dirac and Weyl fermions respectively. Although the surface states from multifold, Dirac, or Weyl fermions can all enhance catalytic performance, their impacts are different due to their differing Fermi arc lengths. See the next section for specific differences.

### 2.4 Catalytic Performance and Fundamental Mechanism

**Figure 4:**
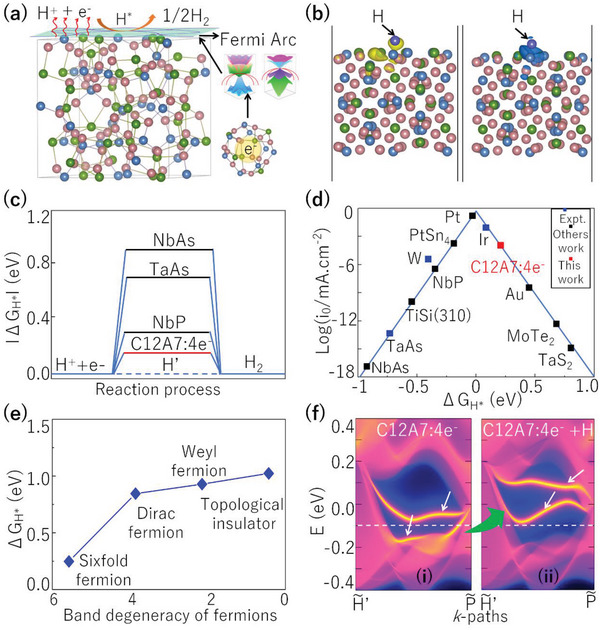
a) Schematic of the mechanism for the inhibited hydrogen poisoning (IHP) process on the (001) surface of C12A7:4e^−^: the long Fermi arcs induced by the SDP and FDP excitations activate the surface for IHP reaction. b) The electron depletion (the left panel) and accumulation (the right panel) during H adsorption on C12A7:4e^−^ surface. The isosurface values are set to 0.0025 e Å^−3^. c) The free energy diagram for hydrogen evolution at a potential *U* = 0 relative to the standard hydrogen electrode at pH = 0. The data for NbP, TaAs and NbAs are taken from ref.[11] d) Volcano plot for IHP of C12A7:4e^−^ in comparison with typical catalysts. The data are taken from refs.[11, 13, 38, 39] e) The quantitative relationship between band degeneracy of different fermions (six‐, Dirac‐, Weyl‐fermions, and topological insulator) and Δ*G*_H*_ on the (001) surface. f) shows the (001) surface states at the specific path of C12A7:4e^−^ without (i) and with (ii) the hydrogen adsorption, respectively.

We study the catalytic performance of C12A7:4e^−^ by estimating the adsorption of hydrogen (H) and examining the chemical process of H_2_ production on the (001) surface, as shown in **Figure** **4**a. This corresponds to the inhibited hydrogen poisoning (IHP) process in ammonia synthesis, which is similar to the HER process discussed for TaAs and PtAl.^[^ ^6^, ^11^ ^]^ We use the change in Gibbs free energy (ΔG_H*_) of hydrogen adsorption, ΔG_H*_, on the catalyst surface to judge the hydrogen evolution reaction. We first make a detailed investigation of the active sites on the (001) surface of electride C12A7:4e^−^. Based on the topology of the lattice and adsorption energy calculations (see Figures S2 and S3 of Supporting Information^[^ ^33^ ^]^), we find the site on the top‐Ca of the surface nanopore cages is the most stable among the three active sites. It is worth noticing that, the excess electrons located at the active nanopore cages mainly contribute to the electronic states of the multifold fermions (See Figure 1d‐e). In addition, we also investigate the potential pathway of H^+^ reduction to H_2_ on the surface of C12A7:4e^−^. Comparing with the Tefel step, the Volmer‐Heyrovsky step has a much lower energy barrier (1.6 eV, see Figures S4 of Supporting Information^[^ ^33^ ^]^), suggesting the Volmer‐Heyrovsky mechanism is dominant for H^+^ reduction to H_2_ on the surface of C12A7:4e^−^. We further estimate the ΔG_H__*_ at the active sites. As shown in Figure 4c,d, the absolute value |ΔG_H*_| is significantly lower than those in Weyl catalysts NbP (0.31 eV), TaAs (0.74 eV), and NbAs (0.96 eV).^[^ ^11^ ^]^ This point has been demonstrated from our further calculations. The sixfold fermions in C12A7:4e^−^ can be artificially transferred into different topological states with lower band degeneracy (Dirac fermions, Weyl fermions, and topological insulators) under strain (Figure 3a), |Δ*G*_H*_| is found to decrease linearly with the increasing band degeneracy (See Figure 4e), indicating the long Fermi arcs in C12A7:4e^−^ play an important role in the catalytic enhancement. As shown by the volcanic curves in Figure 4d, C12A7:4e^−^ sits nearly at the top of the volcanic curve, indicating the high activity of the surface. In Figure 4f, we further compare the variations of the surface states of C12A7:4e^−^ before [Figure 4f (i)] and after hydrogen adsorption [Figure 4f (ii)], and find the long Fermi arcs are robust during the adsorption process, but shift upward as indicated by the surface DOS variation (See Figure S5 of Supporting Information^[^ ^33^ ^]^) due to the electron donation from the *s*‐orbital of the adsorbed hydrogen. This process can be further confirmed from the charge density difference (CDD) in Figure 4b — charge depletion occurs on the surface of C12A7:4e^−^, while charge accumulation happens around the adatom. We estimate the catalytic activity in the bulk, especially in the bulk lattice cages. Our results show Δ*G*_H*_ inside the bulk cages is remarkably larger (absolute value: 0.51 eV) than that on the surface (0.24 eV), indicating that the surface is more active for catalysis. This confirms the importance of the surface state, and explains the recent experimental findings of the enhanced catalytic performance in Ru/C12A7:4e^−^.^[^ ^23^ ^]^

**Figure 5:**
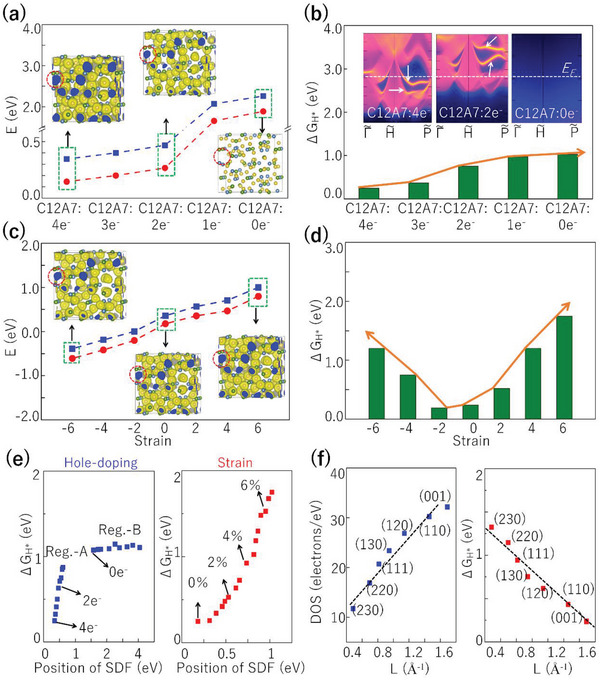
a) The positions of SDP (blue line) and FDP (red line) in C12A7:4e^−^ with different charge states. The insets in (a) provide the ELF maps for C12A7:4e^−^/:2e^−^/:0e^−^, where the isosurface values are 0.65. b) The ΔG_H*_ of the (001) surface in C12A7:4e^−^ with different charge states. The insets in (b) show the corresponding (001) surface states for C12A7:4e^−^/:2e^−^/:0e^−^. c) The energy position of SDP and FDP corresponding to the Fermi level under hydrostatic distortions from −6% to +6%, where negative and positive values denote lattice compression and lattice expansion, respectively. The insets in (c) provide the ELF maps of electride C12A7:4e^−^ under −6%, 0%, and +6% lattice distortions, where the isosurface values are 0.65. d) The change of ΔG_H*_ on the (001) surface of C12A7:4e^−^ under different lattice distortions. e) The change of Δ*G*_H*_ on different positions of SDF by hole‐doping and strain. f) The linear relationship between Fermi arc length (L) of different surfaces and the corresponding surface DOSs (the left panel) and the catalytic Δ*G*_H*_ (the right panel).

The effect of multifold fermions and surface Fermi arcs on catalytic performance can be directly observed when surface Fermi arc states are removed or shifted to a higher energy level. This scenario can be realized by artificially annihilating the excess electrons (via hole doping) in C12A7:4e^−^. As shown in **Figure** **5**a and Figure S6, Supporting Information,^[^ ^33^ ^]^ by gradually annihilating excess electrons in the system, the interstitial electrons are reduced. After four excess electrons have been annihilated, the system no longer contains interstitial electrons. The SDP and FDP remain during this process because the symmetry is unchanged. However, both of them will move away from the Fermi level towards extremely higher energy levels, where SDF moves from 0.346 to 2.26 eV and FDP moves from 0.146 to 1.886 eV, as shown in Figure 5a and Figure S7, Supporting Information,^[^.^[^ ^33^ ^]^ Correspondingly, as shown in Figure 5b, Δ*G*_H*_ has obviously increased from 0.248 to 1.025 eV. Such reduction of the catalytic performance can be attributed to the shift of surface Fermi arc states in the system. The insets of Figure 5b compare the surface states within |*E* − *E*_F_| = 0.5 eV for native C12A7:4e^−^, and the cases with annihilating two (C12A7:2e^−^) and four (C12A7:0e^−^) excess electrons. One can clearly see Fermi arcs at the Fermi level for C12A7:4e^−^. In C12A7:2e^−^, although surface Fermi arcs exist, they are located above the Fermi level. For C12A7:0e^−^ or :1e^−^, no conductive surface states are found for energies ± 0.5 eV. The system becomes an insulator, it is not an electrocatalyst and the Δ*G*_H*_ will remain unchanged, see the left plane of Figure 5e. The catalytic performance strongly correlates with the surface states, as shown in Figure 5b, which follows: C12A7:4e^−^ (0.248 eV) < C12A7:2e^−^ (0.714 eV) < C12A7:0e^−^ (1.025 eV).

We further confirm this correlation arises from the surface Fermi arcs, rather than the excess electrons, by applying hydrostatic distortions to the C12A7:4e^−^ lattice. We find that the electride properties are nearly unchanged — the four excess interstitial electrons are preserved during lattice distortion as shown in the ELF maps in Figure 4c and in Figure S8, Supporting Information.^[^ ^33^ ^]^ However, the multifold fermions are strain sensitive. As shown in Figure 5c and Figure S9, Supporting Information,^[^ ^33^ ^]^ the energy positions for SDP and FDP move away from the Fermi level under both lattice compression and expansion. For example, the FDP (SDP) is increased from −0.61 eV to 0.796 eV (−0.39 to 1.02 eV) under a strain of ±6%. Correspondingly, ΔG_H*_ at the (001) surface is increased from 0.248 eV to 1.241 eV (1.75 eV) under strain of −6% (+6%), see Figure 5d. This explicitly demonstrates that the excellent catalytic performance indeed originates from multifold fermions and corresponding long surface Fermi arcs near the Fermi level rather than the presence of excess electrons. It is worth to noting, the strain has a more profound impact on Δ*G*_H*_ than the hole doping, due to the combined effects of the strain‐induced shifts of multifold fermions and the strain itself (See the right plane of Figure 5e and Figure S10 of Supporting Information^[^ ^33^ ^]^). More explicit evidence can be seen from the quantitative relationship between the Fermi arc and the catalytic performance. Our calculations show the Fermi arcs for the multifold fermions can exist in several surfaces including (001), (110), (111), (120), (130), (220), (230) (See Figure S11 of Supporting Information^[^ ^33^ ^]^). Interestingly, the length of Fermi arcs (L) on these surfaces and the catalytic Δ*G*_H*_ exhibits a linear relationship, which follows: Δ*G*_H*_ = −0.079 L + 1.382 (See the right panel of Figure 5f). The origin of the quantitative relationship can be understood as follows. The presence of Fermi arcs can lead to an excellent surface conductivity, which is highly important for catalytic enhancement. The surface DOSs of C12A7:4e^−^ indeed show a positive correlation with the length of Fermi arcs (See the left panel of Figure 5f and Figure S12, Supporting Information^33^). (The specific values are shown in Tables SIII and IV of Supporting Information^[^ ^33^ ^]^)

In addition, we take electride Li_12_Al_3_Si_4_:5e^−^ as a contrast comparison, which has the same crystal space group with C12A7:4e^−^. Li_12_Al_3_Si_4_:5e^−^ also has excess electrons trapped in interstitial sites and the multifold fermions (Figures S13 and S14, Supporting Information^[^ ^33^ ^]^). However, unlike electride C12A7:4e^−^, the FDP and FDP in electride Li_12_Al_3_Si_4_:5e^−^ are far away from the Femi level and their surface states are unlikely to contribute to the conducting active in the system. As a result, Li_12_Al_3_Si_4_:5e^−^ has a much higher ∆*G*_H*_ on the (001) surface than C12A7:4e^−^ (1.90 eV vs 0.24 eV) (Figures S13 and S14, Supporting Information^[^ ^33^ ^]^). These results strongly indicate that the catalytic enhancement in electride C12A7:4e^−^ originates from the multiple‐fold‐fermions‐induced Fermi arc on the (001) surface, rather than the excess electrons.

Before closing, we have following remarks. First, we would like to further clarify the logic of topological properties relating to the catalytic activity. In electride C12A7:4e^−^, the catalytic active site is determined as the surface nanocages of the lattice. The excess electrons locating at these active nanocages mainly contribute to the electronic states of the multifold fermions, which characterize with linear band crossings near the Fermi energy and the presence of long Fermi arcs on the surface. Both the linear band crossings and nontrivial surface states can effectively induce high carrier mobility in topological materials.^[^ ^40^, ^41^, ^42^, ^43^, ^44^ ^]^ Indeed, the carrier mobility of C12A7:4e^−^ has been experimentally verified as high as 200 cm^2^ V^−1^ S^−1^ at the room temperature.^[^ ^34^ ^]^ Besides, the Fermi arcs can also contribute high electronic density of states on the surface. Our calculations show the electronic density at the Fermi level for the (001) surface is 2.7 times larger than that of the bulk (See Figure S15 of Supporting Information^[^ ^33^ ^]^). A similar phenomenon has also been found in other topological materials.^[^ ^10^, ^14^, ^45^, ^46^ ^]^ Most importantly, the multifold fermions and Fermi arcs have been verified to be highly relative with the catalytic activity Δ*G*_H*_, where the position of multifold fermions, the intensity of surface DOSs, and the length of Fermi arcs are found to linearly corelate with the catalytic performance (See Figures 3, 4, 5). Benefiting from the multifold fermions and the long surface Fermi arcs, electride C12A7:4e^−^ naturally combines high carrier mobility, high surface electronic densities and low Δ*G*_H*_, which are all crucial indexes determining its high catalytic activity.

Second, besides the clarification of the mechanism of IHP process in electride C12A7:4e^−^, our above calculations have also estimated the performance of C12A7:4e^−^ on the electrocatalytic process of hydrogen evolution. The results suggest the material is theoretically potential as a catalyst for electrocatalytic hydrogen evolution with high carrier mobility, high surface electronic densities and low ΔG_H*_. Besides, C12A7:4e^−^ was found to be able to initiate anionic electron transfer in the water‐splitting process.^[^ ^47^ ^]^ and promote photocatalytic H_2_ production.^[^ ^48^ ^]^ It is also promising to be used for thermocatalytic hydrogen evolution due to its excellent thermal stability (the decompose temperature is as high as ≈1500 K).^[^ ^34^ ^]^

Third, we remark on the long surface Fermi arcs in C12A7:4e^−^, which is responsible for the high catalytic activity in C12A7:4e^−^. From the theoretical point of view, the lengths of these Fermi arcs can be directly measured from the *k*‐space separation of the multifold nodes (See Figure S11 of Supporting Information^[^ ^33^ ^]^). A similar length measurement of Fermi arc has been applied in Weyl semimetals previously.^[^ ^49^ ^]^ For the experimental point of view, the Fermi arcs of the C12A7:4e^−^ are quite promising to be directly observed by using bulk‐ and surface‐sensitive angle‐resolved photoemission spectroscopy (ARPES) based on the following facts: i) C12A7:4e^−^ is very stable at room temperature and low temperature, and its high‐quality single‐crystal samples have already been prepared since 2003^[^ ^34^, ^50^, ^51^ ^]^; ii) The multifold fermions locate very close to the Fermi energy and surface band structure shows the long surface Fermi arcs are very clear and are well separated from the bulk states; iii) Several topological materials with a similar structure containing multiple fermions and Fermi arcs have already been successfully observed by ARPES experiments.^[^ ^40^, ^52^, ^53^, ^54^ ^]^

## 3 Conclusions

In conclusion, we revealed the presence of surface Fermi arc states and multifold fermions (SDP and FDP) in electride C12A7:4e^−^. The multifold fermions are protected by symmetry and exhibit novel surface states with extremely long Fermi arcs nearly traversing the entire (001) surface of the Brillouin zone. These Fermi arcs make the (001) surface highly electronically active, and lead to a relatively low Δ*G*_H*_ (0.24 eV) during the IHP catalytic process. Notably, this Δ*G*_H*_ is lower than that of most topological quantum catalysts proposed previously. Our work provides a new understanding of the catalytic nature in electrode‐based catalysts by analyzing their topological quantum states. We, for the first time, explicitly identified the important role of surface Fermi arcs in heterogeneous catalysis, providing a deeper understanding of topological catalysis and clarifying the experimental debates.

## 4 Experimental Section

The first‐principles calculations were performed by using the Vienna *ab*‐initio simulation package (VASP).^[^ ^55^, ^56^ ^]^ For electride C12A7:4e^−^, we used the generalized gradient approximation (GGA) of Perdew—Burke–Ernzerhof (PBE) method.^[^ ^57^ ^]^ For electride C12A7:4e^−^, the cutoff value of plane wave kinetic energy was adopted as 400 eV, the Brillouin zone (BZ) was sampled with Γ‐centered *k*‐point mesh of 11 × 11 × 11 for both structural optimization and self‐consistent calculations. The energy convergence criteria were chosen as 10^−5^eV. For electride C12A7:4e^−^ and electride Li_12_Al_3_Si_4_:5e^−^ the surface states were calculated by using Wannier functions and by using the iterative Green's function method as implemented in the WannierTools package.^[^ ^58^ ^]^ In addition, in order to calculate the surface activity of electride C12A7:4e^−^, we set up a vacuum layer, ≈20 Å. For electride C12A7:4e^−^, C12A7:4e^−^•H with a vacuum layer, the Brillouin zone (BZ) was sampled with Γ‐centered *k*‐point mesh of 11 × 11 × 1 for both structural optimization and self‐consistent calculations. For electride C12A7:4e^−^, band representations (BRs) can be can be generated by the code pos2aBR.^[^ ^36^, ^37^ ^]^ Besides, we use the same calculation methods for electride Li_12_Al_3_Si_4_:5e^−^. Besides, the electronic band structures with SOC are shown in Figure S16, Supporting Information.

Here, the hydrogen evolution reaction (HER) refers to the use of catalysts to produce hydrogen by electrochemical methods. Inhibiting hydrogen poisoning (IHP) means that electrides can promote the adsorption and release of hydrogen in ammonia synthesis. Although these two processes are different, the prevention mechanism of hydrogen poisoning is similar to that of surface H‐adsorption and exhalation in HER. To obtain the Gibbs free energy ($\Delta G_{\left(H^{*}\right)}$) of IHR on the (001) surface and in cages of C12A7:4e^−^, we adopt the method proposed by Norskov et al.^[^ ^59^ ^]^ the process can be written as:

(12) $$H^{+}+e^{-}\to H^{\#}$$

Here, $H^{\#}$ is intermediate products. Then,

(13) $$H^{\#}\to \frac{1}{2}H_{2}$$

To get the $\Delta G_{H^{*}}$ of the IHP on the (001) surface of C12A7:4e^−^, we first calculate the energy difference between the three terms as follows:

(14) $$\Delta G_{H^{*}}\;=\Delta E_{H}+\Delta E_{ZPE}-T\Delta S_{H}$$

Here, Δ*E_H_* is the adsorption energy, Δ*E_ZPE_* is the change of the zero‐point energy, and Δ*S_H_* is the change if the entropy of the adsorbed hydrogen on the (001) surface. First, Δ*E_H_* can be written as:

(15) $$\Delta E_{H}=\;\left[E_{\left(\left[C12A7\right]+nH\right)}-E_{\left(\left[C12A7\right]\right)}+\frac{n}{2}E_{\left(H_{2}\right)}\right]$$

Among, *E*_([*C*12*A*7] + *nH*)_, *E*_([*C*12*A*7])_, $E_{\left(H_{2}\right)}$ are the energies C12A7:4e^−^ with hydrogen adsorbed on (001) surface, the electride C12A7:4e^−^, and the H_2_, respectively.

Second. Δ*E_ZPE_* are 0.022 eV, which agrees well with previously calculated results.^[^ ^59^ ^]^ Finally, Δ*S_H_* can be written as

(16) $$\Delta S_{H}\approx {-}_{2}^{1}S_{H}^{0}$$

Therefore, for electride C12A7:4e^−^, the $\Delta G_{H^{*}}$ of the HER on the (001) surface can be written as:

(17) $$\Delta G_{H^{*}}=\;\Delta E_{H}+0.02eV$$

## Conflict of Interest

The authors declare no conflict of interest.

## Supporting information

Supporting Information

## Data Availability

Research data are not shared.
