# Supplementary material for: Multifold Fermions and Fermi Arcs Boosted Catalysis in Nanoporous Electride 12CaO·7Al2O3
Source: Adv Sci (Weinh). 2022 Dec 27;10(6):2205940. doi: 10.1002/advs.202205940 (PMC9951387; doi:10.1002/advs.202205940)
Supplement: Supplementary file 1 — Supporting Information [file ADVS-10-2205940-s001.pdf]

Supporting Information

for

Multifold Fermions and Fermi Arcs Boosted Catalysis in Nanoporous Electride $12CaO{\cdot}7Al_2O_3$

Weizhen Meng, [1,2] Xiaoming Zhang, *, [1,2] Ying Liu, [1,2] Xuefang Dai, [1,2] Guodong Liu, *, [1,2] Yuantong Gu, [3] E. P. Kenny, [3] and Liangzhi Kou *, [3]

[1] State Key Laboratory of Reliability and Intelligence of Electrical Equipment, Hebei University of Technology, Tianjin 300130, China.

[2] School of Materials Science and Engineering, Hebei University of Technology, Tianjin 300130, China.

[3] School of Mechanical, Medical and Process Engineering, Queensland University of Technology, Garden Point Campus, QLD 4001, Brisbane, Australia.

*Email: zhangxiaoming87@hebut.edu.cn, gdliu1978@126.com, liangzhi.kou@qut.edu.au.

Table of contents

1. The application of topological quantum chemistry in electride C12A7:4e$^-$ (Table SI)

2. Irreducible representations for bands in electride C12A7:4e$^-$ (Fig. S1, Table SII)

3. Active sites and hydrogen production pathways in electride C12A7:4e$^-$ (Figs. S2-4)

4. Surface DOSs before and after hydrogen adsorption in electride C12A7:4e$^-$ (Fig. S5)

5. Electronic structures and ELFs of electrides C12A7:4e$^-$/3e$^-$/2e$^-$/1e$^-$/0e$^-$ (Fig. S6 and S7)

6. Electronic structures and ELFs of hydrostatic strain in electride C12A7:4e$^-$(Figs. S8, S9)

7. The different of ΔG_{H*} between doped holes and strain in electride C12A7:4e$^-$(Fig. S10)

8. Fermi arcs of different surfaces in electride C12A7:4e$^-$(Fig. S11)

9. Surface DOSs and Fermi arc lengths of different surfaces in electride C12A7:4e$^-$ (Fig. S12)

10. Specific values of SDP, FDP and ΔG_{H*} (Tables SIII and IV)

11. Crystal structure and catalytic performance for elelctride $Li_{12}Al_3Si_4$:5e$^-$ (Figs. S13 14, Table SV)

12. Surface electronic density in C12A7:4e$^-$ (Fig. S15)

13. The electronic band structure with spin-orbital coupling (SOC) (Figs. S16)

1. The application of topological quantum chemistry (TQC) in electride C12A7:4e$^-$:

Based on the topological quantum chemistry (TQC) [1], band representations (BRs) of all atoms (Ca, Al, O) and the interstitial electrons are shown in Table SI. Here, BRs (A@B) represent Wyckoff sites (A) and irreducible representations (B) [2]. We find that the BRs of the interstitial electrons are different from that of the atoms, as shown in Table SI. Besides, we find that the bands near the Fermi level (-0.3 ~ 0.3 eV) origins from interstitial electrons with *s*-like orbital characteristics.

Table SI The Wyckoff positions (A), configured orbitals (Conf.), irreducible representations (B) and band representations (BRs) for different atoms and interstitial electrons in C12A7:4e$^-$.

Atoms	Ca	Al1	Al2	O1	O2	Interstitial
Wyckoff sites (A)	24*d*<br>(0.924, 0, 0.25)	12*a*<br>(1, 0.25, 0.125)	16*c*<br>(1.02, 1.02, 1.02)	16*c*<br>(0.935, 0.935, 0.935)	48*e*<br>(0.9, -0.19, 0.713)	12*b*<br>(0.889, 0, 0.75)
Conf.	$3p^6$, $4s^2$	$3s^2$, $3p^1$	$3s^2$, $3p^1$	$2s^2$, $2p^4$	$2s^2$, $2p^4$	*s*-like
Irreducible representations (B)	s: $\Gamma1$ (A_1)<br>p_x: $\Gamma2$ (B_1)<br>p_y: $\Gamma4$ (B_2)<br>p_z; $\Gamma3$ (A_2)	s: $\Gamma1$ (A_1)<br>p_x: $\Gamma4$ (B_1)<br>p_y: $\Gamma1$ (^2E)<br>p_z; $\Gamma3$ (^1E)	s: $\Gamma1$ (A_1)<br>p_x: $\Gamma1$ (A_1)<br>p_y: $\Gamma1$ (^2E)<br>p_z; $\Gamma3$ (^1E)	s: $\Gamma1$ (A_1)<br>p_x: $\Gamma1$ (A_1)<br>p_y: $\Gamma1$ (^2E)<br>p_z; $\Gamma3$ (^1E)	s: $\Gamma1$ (A_1)<br>p_x: $\Gamma4$ (B_1)<br>p_y: $\Gamma1$ (^2E)<br>p_z; $\Gamma3$ (^1E)	
BRs: A@B	$A_1/B_1/A_2/B_2$ @ 48*e*	$A_1/B_1/{}^2E/{}^1E$ @12*a*	$A_1/{}^2E/{}^1E$@ 16*c*	$A_1/{}^2E/{}^1E$@ 16*c*	$A_1/B_1/{}^2E/{}^1E$ @48*e*	A_1@12*b*

2. Irreducible representations for bands in electride C12A7:4e$^-$:

Based on symmetry analysis, all irreducible representations (IRs) of space group 220 (No. 220) are shown in Table S II. Then, we calculate the IRs for C12A7:4e$^-$ along *k*-paths H-Γ-P-H, as show in Table S II. Besides, the enlarged band structures along *k*-paths H-Γ-P-H are displayed in Figure S 1 (a, b). Specifically, we find that the bands along the Γ-H path contains four singly degenerate bands and one doubly degenerate band (Γ_2+Γ_4), the bands along the H-N path contains three doubly

degenerate bands (Γ_1+Γ_2). Besides, the little group at H point belongs to T_d point group, which is generated by $\left\{S_{4x}^{+}\middle|\frac{1}{2}00\right\}$, $\left\{M_{110}\middle|\frac{1}{2}00\right\}$, $\left\{C_{3,1\overline{1}\overline{1}}^{-}\middle|1\frac{1}{2}\frac{1}{2}\right\}$ and together with time-reversal symmetry. There are two three-dimensional irreducible representations (IRs), namely Γ_6 and Γ_7, at this point, they can be connected by time-reversal symmetry, leading to a sixfold degenerate point. Similarly, there exists a doubly degenerate band (Γ_3) and two singly degenerate bands on the Γ-P and P-H paths, respectively. Furthermore, P point is not a time-reversal invariant momentum, such that the effective Hamiltonian at this point does not respect the time-reversal symmetry. In addition, there are four independent symmetry operations at this point, namely $\left\{S_{4x}^{+}\middle|\frac{1}{2}00\right\}$, $\left\{C_{3,1\overline{1}\overline{1}}^{+}\middle|1\frac{1}{2}\frac{1}{2}\right\}$, $\left\{C_{2y}\middle|0\frac{1}{2}\frac{1}{2}\right\}$, $\left\{C_{2x}\middle|\frac{3}{2}\frac{3}{2}0\right\}$. Figure S 1 (c, d) show the 3D dispersion bands of SDP and FDP, which agree well with the results of our symmetry analysis.

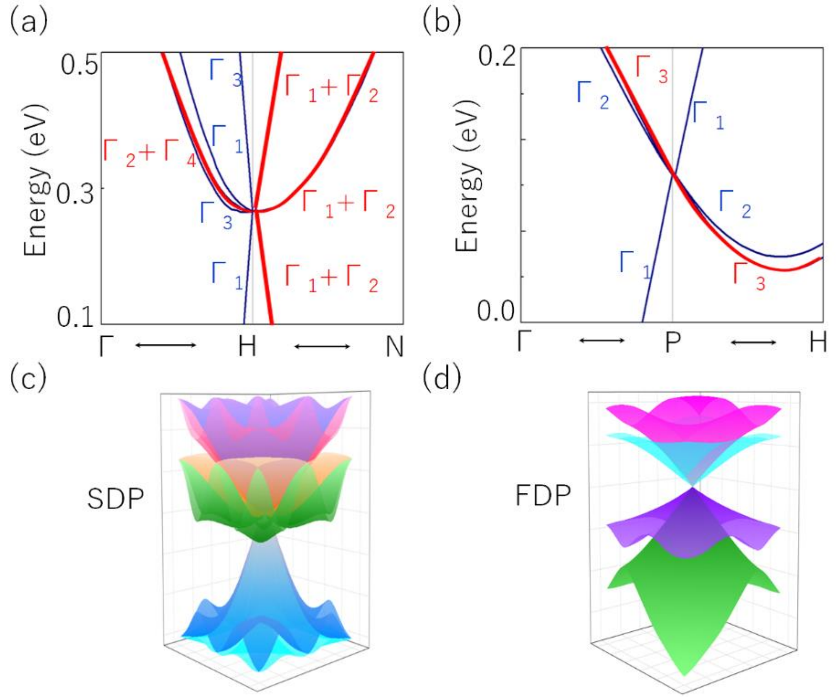

Figure S1 (a) and (b) The enlarged band structures along with the irreducible representations near P_1 and P_2. The red and blue bands show doubly and singly degenerate band, respectively. (c) and (d) The three-dimensional (3D) dispersion of SDP and FDP, respectively.

Table SII The Irreducible representations (IRs) of space group 220, and the IRs in the energy range -2.0 eV to 1.0 eV in electride C12A7:4e⁻. The IRs are given in order of increasing energy eigenvalues.

k-paths	Γ-H	H	Γ-P	P	P-H
No. 220	Γ_1, Γ_2, Γ_3, Γ_4	Γ_1, Γ_3, Γ_2, Γ_4, Γ_5	Γ_1, Γ_2, Γ_3	Γ_1, Γ_2, Γ_3	Γ_1, Γ_2, Γ_3
$C_{12}A_7$:4e⁻ (E_f=-2~1eV)	Γ_1, Γ_3 Γ_2+Γ_4 (two-fold), Γ_1, Γ_3	Γ_6+Γ_7 (six-fold)	Γ_3 (two-fold), Γ_2, Γ_1	Γ_4 (four-fold)	Γ_3 (two-fold), Γ_2, Γ_1

3. Active sites and hydrogen production pathways in electride C12A7:4e⁻:

In electride C12A7:4e⁻, the nanopore cages are interconnected to form the entire skeleton of the lattice (see Fig. 1a). Figure S2(a) shows the topology of nanopore cages along the [001] direction, ordered as A, B, C layers, where the nanopore cages of B-layer (B-L) are exposed to (001) surface. In other words, the (001) surface is made up of numerous B-L nanopore cages connected to each other. Therefore, we only need to consider the potential active sites on a B-L nanopore cage, similar consideration is also applied in a previous work where Au adsorption on electride C12A7:4e⁻ was studied. As shown in Fig. S2(b) below, six-potential active sites are considered on the nanopore cage, namely top-Ca atom (S_1), hollow-Ca atom (S_2), O atom (S_3), vacancy (S_4), Al (S_5) and bridge of Ca-O atoms (S_6), respectively. The calculated results find that the adatom at the S_2, S_3 and S_6 active sites are unable, which finally move to S_4 active site. On the contrary, the S_1 and S_5 active sites are relatively stable, as shown in Fig. S3 (a-d) below. Based on the DFT calculation, the final adsorption energy (E_{ads}) relationship of the three stable active sites is S_1 (-0.22 eV) > S_4 (-0.175 eV) > S_6 (-0.022 eV), indicating that S_1 (namely top-Ca) is the most stable active site.

It is well known that, the pathway of H^+ reduction to H_2 usually takes two mechanisms, namely Volmer-Tafel and Volmer-Heyrovsky. For the Tafel step in electride C12A7:4e⁻, two H* at stable adsorption sites on the (001) surface form the

final hydrogen molecule (H_2), namely H* + H* → H_2 (See Fig. S4a below). For Heyrovsky step of electride C12A7:4e$^-$, an H* at stable adsorption sites on the (001) surface combines with an itinerant H^+ in the water to from the final H_2, namely, H* + H^+ + e^- → H_2 (See Fig. S4b below). Our calculations show that the Tafel step in electride C12A7:4e$^-$ require to overcome an extremely high barrier to form H_2 (~5.78 eV), due to the relatively large distance between the two stable adsorption sites (~12 Å). On the contrary, the Heyrovsky step of in electride C12A7:4e$^-$ only needs to overcome the 1.6 eV barrier to form H_2 (See Fig. S4c below). These results suggest that the Volmer-Heyrovsky mechanism is dominant for H^+ reduction to H_2.

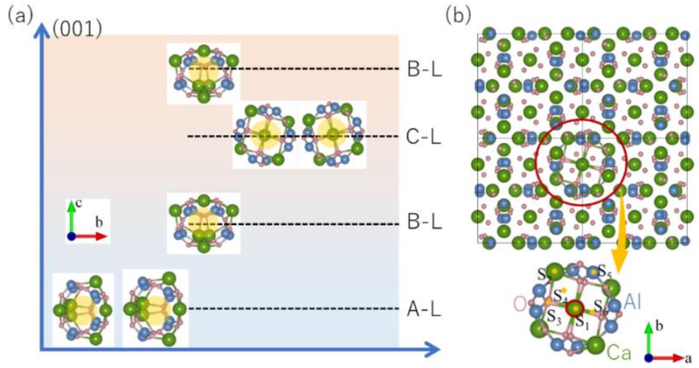

Figure S2 (a) Ordering of the lattice nanopore cages (not to scale) along (001) direction for electride C12A7:4e$^-$. (b) The 2×2×1 supercell structure of electride C12A7:4e$^-$. The side view of a single nanopore cage, where S_1-S_6 are the corresponding possible active sites.

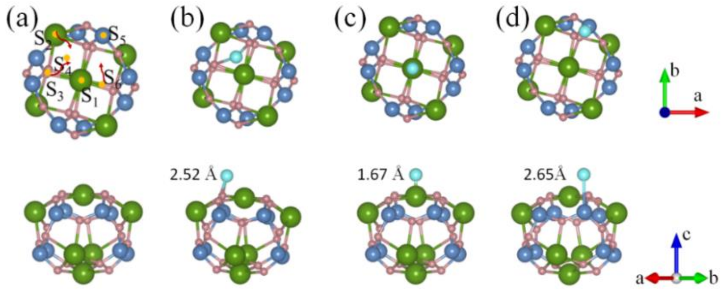

Figure S3 (a) The side view and the top view of a single nanopore cage, where S_1-S_6 are the corresponding possible active sites. (b) – (c) The final optimized structures of C12A7:4e$^-$/H at the active sites S_4, S_1 and S_5.

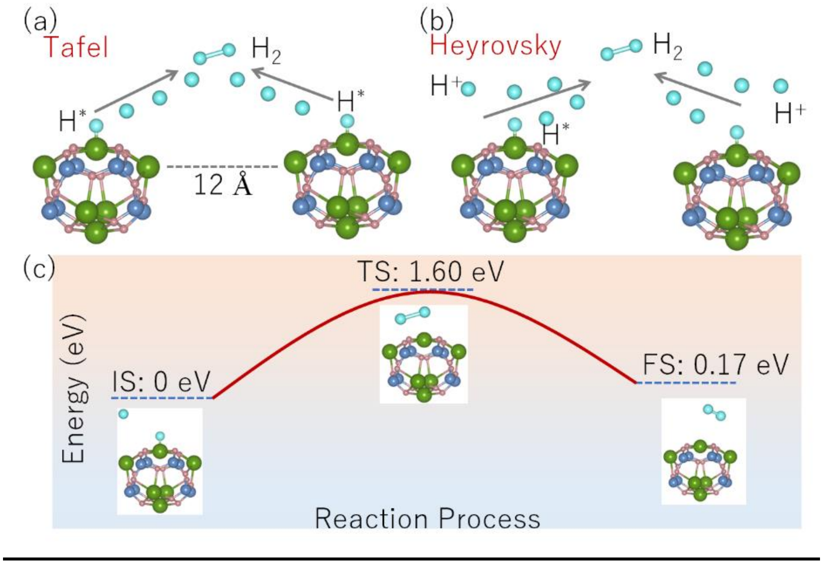

Figure S4 The pathway of H^+ reduction to H_2 usually takes two mechanisms, namely Volmer-Tafel (a) and Volmer-Heyrovsky (b) steps, respectively. (c) The pathway of H^+ reduction to H_2 in the Heyrovsky step needs to overcome barrier for electride C12A7:4e$^-$, where IS, TS and FS represent the initial, transition and final states, respectively.

4. Surface DOSs before and after hydrogen adsorption in electride C12A7:4e$^-$

We further check the upshift of the Fermi arcs by comparing the surface density of states (DOS) before and after hydrogen adsorption. As shown in Figure S2 below, we can clearly find the DOS peak near the Fermi level shift towards to higher energy.

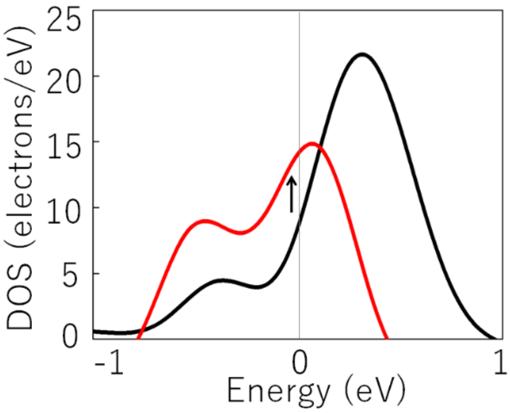

Figure S5 The surface density of states (DOS) of C12A7:4e$^-$ before (the red line) and after (the black line) hydrogen adsorption.

5. Electronic structures and ELFs of elelctrides C12A7:4e$^-$/3e$^-$/2e$^-$/1e$^-$/0e$^-$:

Here, the excess electrons of interstitial sites are annihilated by doping holes, and the corresponding electronic structures of electrides C12A7:4e$^-$, C12A7:3e$^-$, C12A7:2e$^-$, C12A7:1e$^-$, C12A7:0e$^-$ are shown in Figure S 4 (a-c). We find that the electronic structures of C12A7:4e$^-$, C12A7:3e$^-$, C12A7:2e$^-$ show metal feature. However, the electronic structures of C12A7:1e$^-$, C12A7:0e$^-$ show semiconductor feature. The SDP and FDP at H and P points do not disappear because the symmetry does not change. Besides, with the reduction of excess electrons (:4e$^-$ → :0e$^-$), SDP and FDP gradually move away from the Fermi level. In addition, we also show the ELF of C12A7:4e$^-$, C12A7:3e$^-$, C12A7:2e$^-$, C12A7:1e$^-$, C12A7:0e$^-$, respectively. [See Figure S 3 (a, b)]. We find that the excess electrons are gradually annihilated.

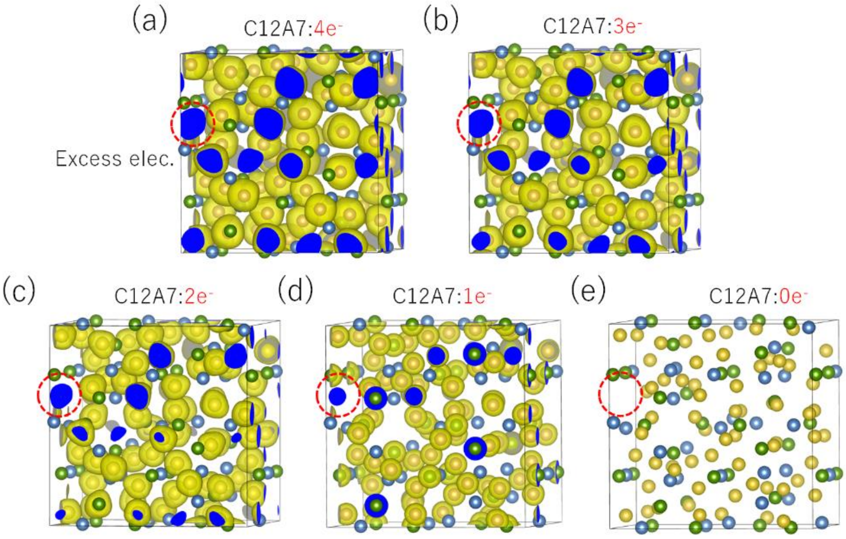

Figure S6 (a-e) show the electron localization function (ELF) of electride C12A7:4e$^-$, C12A7:3e$^-$, C12A7:2e$^-$, C12A7:1e$^-$, C12A7:0e$^-$, respectively. The isosurface values set as 0.65e/Bohr^{-3}

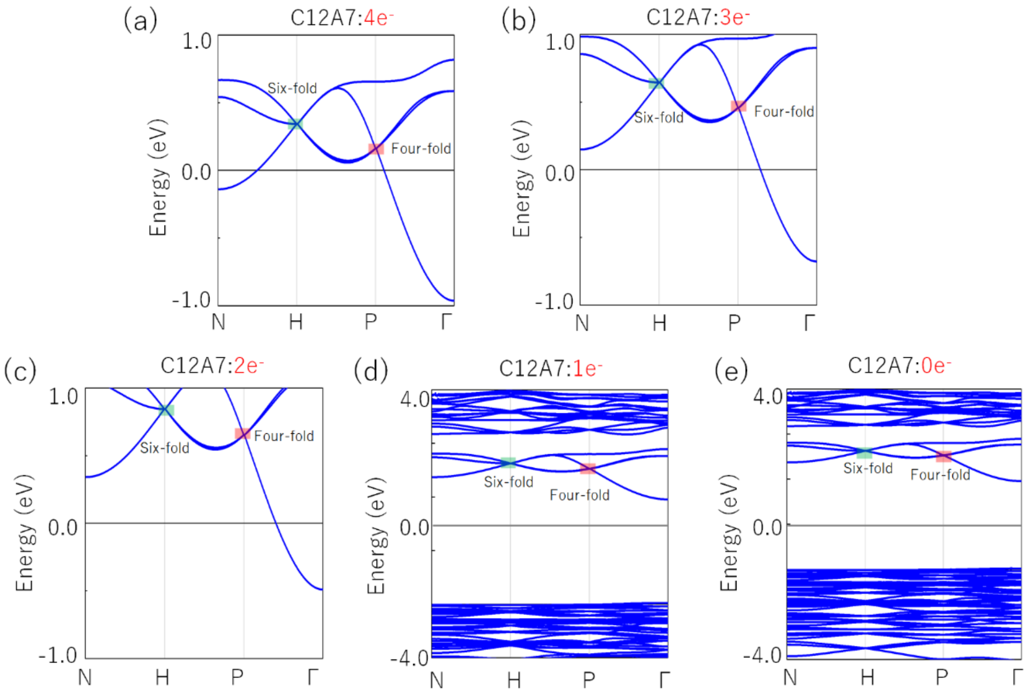

Figure S7 (a-e) show the electronic structures of C12A7:4e$^-$, C12A7:3e$^-$, C12A7:2e$^-$, C12A7:1e$^-$, C12A7:0e$^-$, respectively. The green and red shades represent SDP and FDP on high symmetry points H and P, respectively.

<u>6. Electronic structures and ELFs of hydrostatic distortions in electride C12A7:4e$^-$:</u>

As we all know, the regulation of hydrostatic distortions does not change the space symmetries in electride C12A7:4e$^-$, thus SDP and FDP are not broken. Figure S 6 (a) and (b) show the electronic structures of hydrostatic distortions (-6% ~ 6%) in C12A7:4e$^-$. We find that the SDP and FDP gradually move up the Fermi level under hydrostatic tensile strain (0% ~ 6%). On the contrary, the SDP and FDP gradually move down the Fermi level under hydrostatic compressive strain. Besides, the electronic structure of -4% and 6% is closed to semiconductors. Figure S 5 shows the ELFs of C12A7:4e$^-$ of hydrostatic distortions (namely, -6 % ~ 6 %). We find that the properties of the electrides are not sensitive to strain. In other words, the excess electrons are not annihilated under hydrostatic distortions in C12A7:4e$^-$.

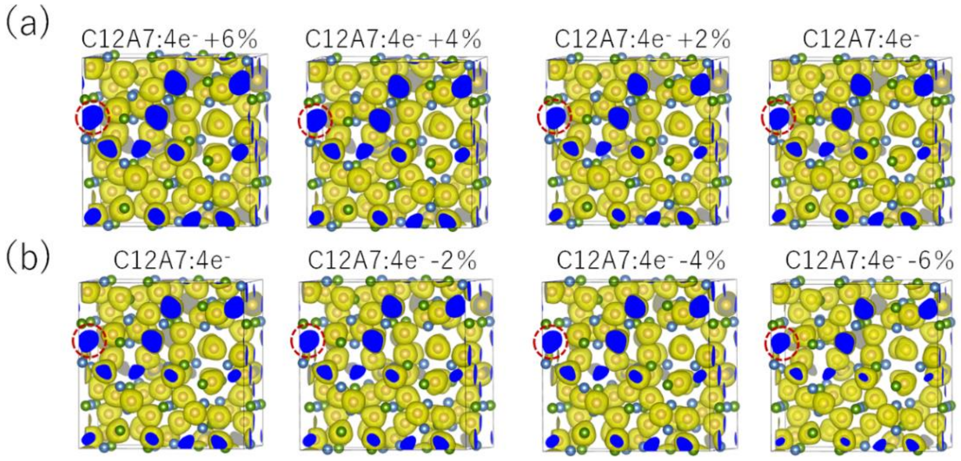

Figure S8 (a) and (b) show the electron localization function (ELF) of electride C12A7:4e$^-$ under different hydrostatic distortions with the isosurface values set as 0.65, respectively.

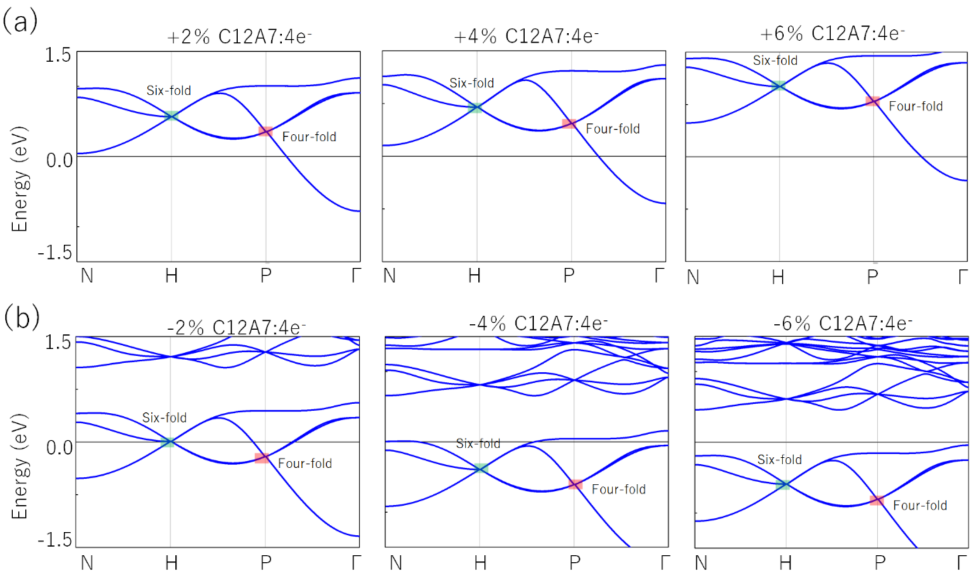

Figure S9 (a) and (b) show the electronic band structures of C12A7:4e$^-$ under different hydrostatic distortions. The green and red shades represent SDP and FDP on points H and P, respectively.

7. The different of ΔG_{H*} between doped holes and strain in electride C12A7:4e$^-$

In Figure S7 (a) below, we show the change of ΔG_{H*} upon the energy of the SDP for the hole doping (the blue line) and the strain (the red line) cases. For the hole doping case, we can find that, at first the ΔG_{H*} monotonically increases with the energy increase of the SDP (region-A). This corresponds to the electrochemical process and the topological Fermi arcs are responsible for the catalytic process. The cases of C12A7:4e$^-$-C12A7:2e$^-$ locate in this region. However, with further hole

doping, the SDP is far away from the Fermi level and the C12A7 system becomes an insulating phase (region-B). This process no longer belongs to electrocatalysis but photocatalysis because the system is insulating. The surface Fermi arc states cannot contribute to the catalytic process thus the ΔG_{H*} becomes nearly unchanged upon the energy shift of the SDP. C12A7:4e^{-}-C12A7:2e^{-} locate in this region. However, for the strain case, we can find ΔG_{H*} always changes during the whole period (see the red line). This indicates the strain itself will affect the ΔG_{H*}. To show this point, we firstly broke the SDP by a 0.1% strain along the [110] direction. Then further apply strain on this lattice. During this period, the surface fermi arc states do not exist. Thus, we can observe the influence on ΔG_{H*} purely from strain. As shown in Figure S7 (b) below, with the increase of strain, we can find ΔG_{H*} would decrease at first but rapidly increase with strain larger than 4%.

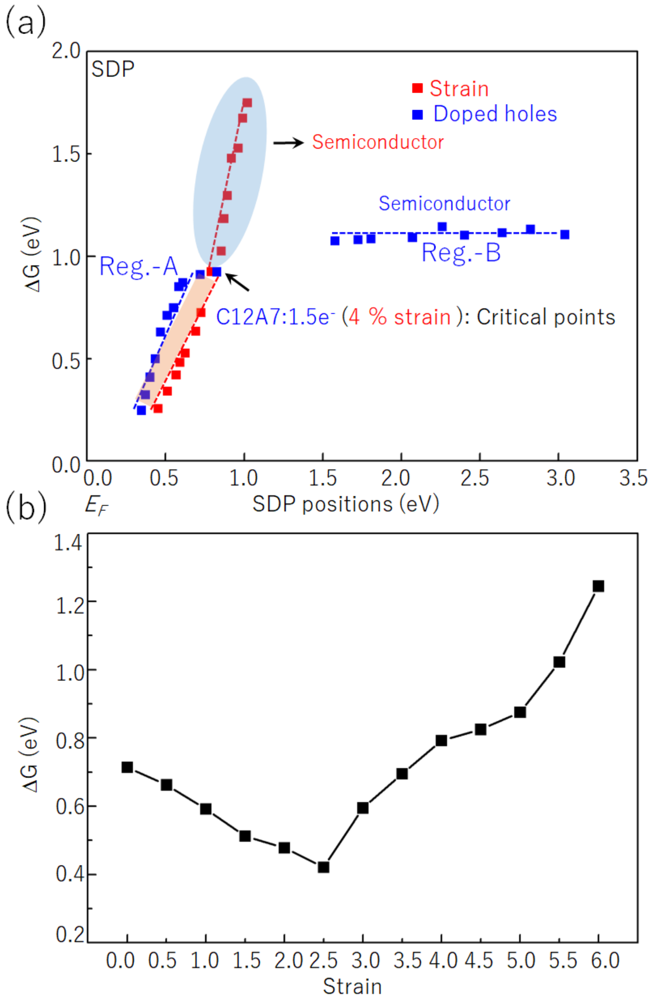

Figure S10. (a) The change of ΔG_{H*} upon different SDF positions under doping holes (blue)

and strain (red). The number of doping holes is 0~6, the range of strain is 0%~8%. (b) The change of ΔG_{H*} at different strain [namely 0 ~ 6%] by breaking the SDP.

8. Fermi arcs of different surfaces in electride C12A7:4e$^-$

The Fermi arcs of the multifold fermions in C12A7:4e$^-$ can be observed on several surfaces including (001), (110), (111), (120), (130), (220), (230) although some are not topologically protected, as shown in Figure S8 (a-f). The lengths of these Fermi arcs are dependent on the surfaces, which can be measured from the *k*-space separation of the multiple-fold nodes. The length measurement of Fermi arc has been applied in Weyl semimetals previously [3], or directly obtained from the ARPES experiments [4,5]

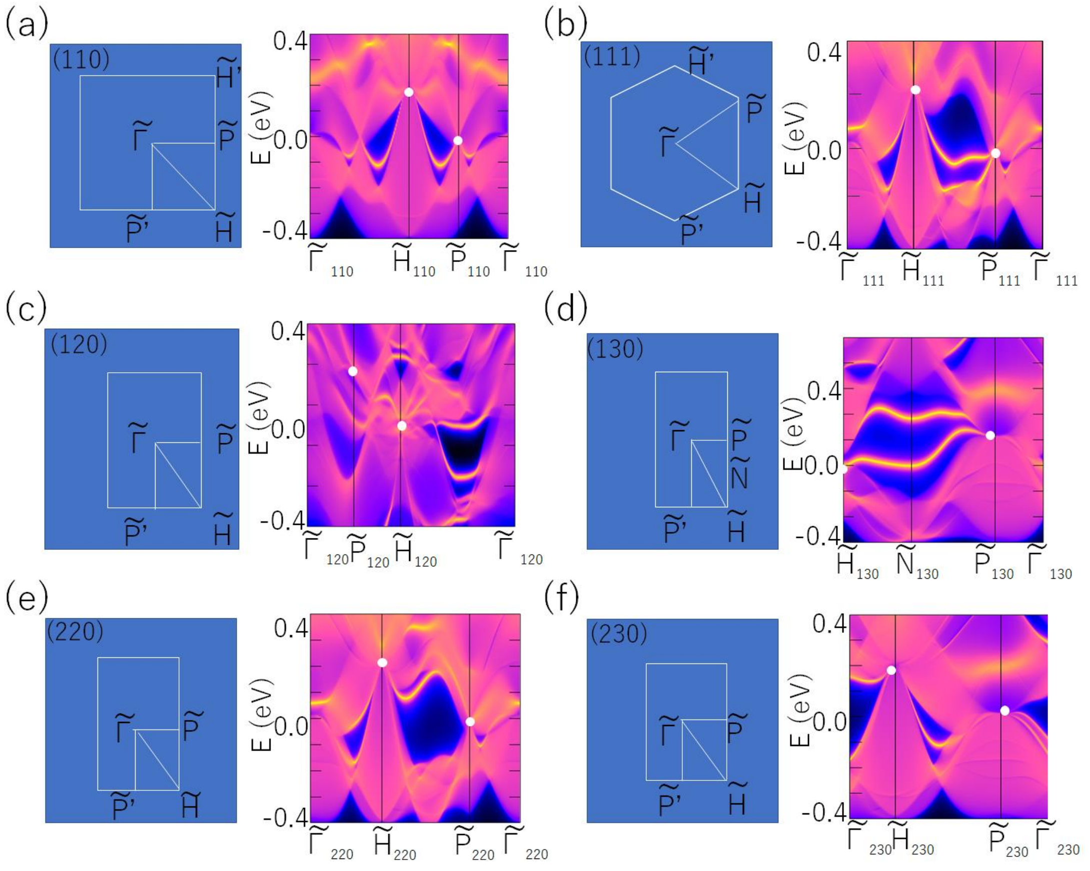

Figure S11 (a) – (f) The k-paths of orbital projection and the surface states and ton (110), (111), (120), (130), (220) and (230) surfaces in electride C12A7:4e$^-$, respectively.

9. DOS of different surfaces in electride C12A7:4e$^-$

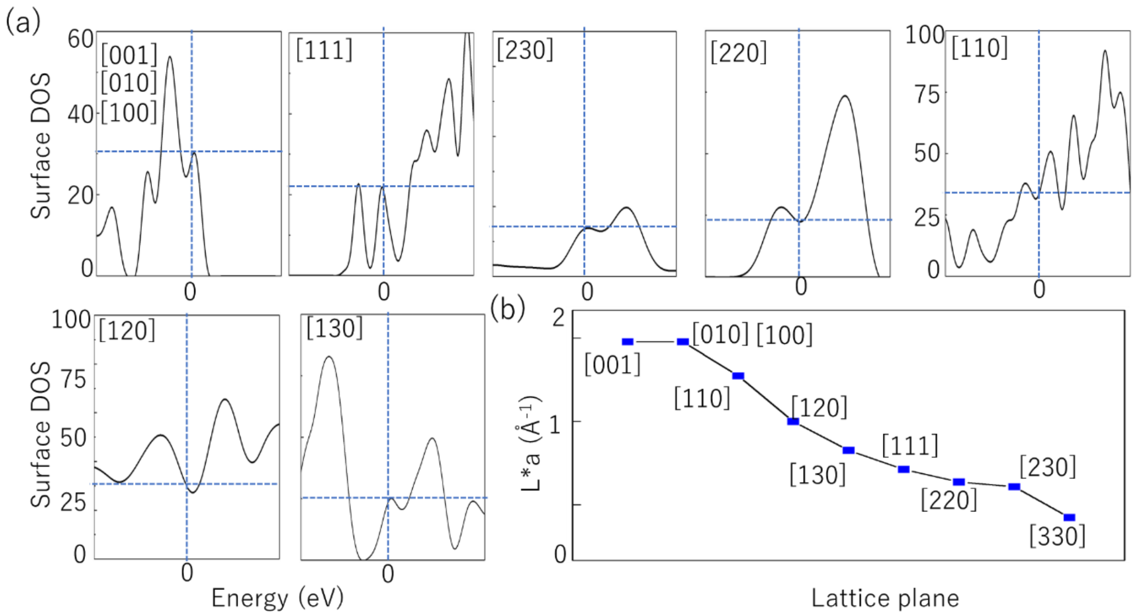

Figure S12 (a) The surface DOSs of different surfaces (e.g., 001, 111, 230…) for electride C12A7:4e$^-$. (b) The lengths of Fermi arc under different planes.

10. Specific values of SDP, FDP and ΔG_{H*}

Table SIII. The energy positions of sixfold and fourfold fermions in electrides C12A7:4e$^-$, C12A7:3e$^-$, C12A7:2e$^-$, C12A7:1e$^-$, C12A7:0e$^-$, and the corresponding catalytic ΔG_{H*}.

Compounds	Sixfold fermion (eV)	Fourfold fermions (eV)	ΔG_{H*} (eV)
C12A7:4e$^-$	0.346	0.146	0.248
C12A7:3e$^-$	0.404	0.201	0.370
C12A7:2e$^-$	0.467	0.267	0.714
C12A7:1e$^-$	2.071	1.654	0.972
C12A7:0e$^-$	2.260	1.886	1.025

Table SIV. The energy positions of sixfold and fourfold fermions of in electrides C12A7:4e$^-$ under different strains (-6% ~ 6%), and the corresponding catalytic ΔG_{H*}.

Strain (%)	Sixfold fermion (eV)	Fourfold fermions (eV)	ΔG_{H*} (eV)
-6	-0.390	-0.610	1.241
-4	-0.190	-0.418	0.752
-2	0.001	-0.203	0.192
0	0.346	0.146	0.248
2	0.567	0.355	0.521

4	0.705	0.467	1.210
6	1.020	0.796	1.750

11. Crystal structure and catalytic performance for elelctride $Li_{12}Al_3Si_4$:5e$^-$:

Figure S10 (a) and (b) show the crystal structures of $Li_{12}Al_3Si_4$. It has a cubic structure and the space group belongs to $I\bar{4}3d$ (No. 220), which has the same crystal space group as Ca12A7:4e$^-$. It also belongs to typical 0D electride. For the optimized crystal structure, the lattice constant yields to be a= b= c= 9.11 Å. The Li atoms occupy the 48*e* Wyckoff sites; Al atoms occupy 12*a* and Si atoms occupy Wyckoff site 16*c*. [See Table S V] Besides, the Li, Al, and Si atoms show +1, +3, and -4 valence states, respectively. As the result, the material totally contains 5 excess electrons for a unit cell, namely $Li_{12}Al_3Si_4$:5e$^-$.

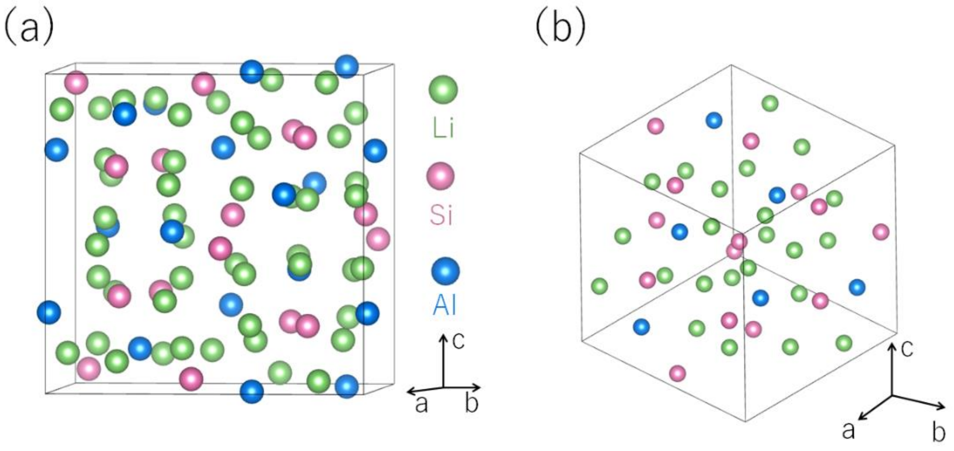

Figure S13 (a) supercell and (b) unit cell for electride $Li_{12}Al_3Si_4$:5e$^-$.

Table SV The Wyckoff positions for electride $Li_{12}Al_3Si_4$:5e$^-$.

Atoms	Li	Al	Si
Wyckoff sites (A)	48*e* (0.124, 0.149, 0.970)	12*a* (0.375, 0.00, 0.25)	16*c* (0.209, 0.209, 0.209)

As shown in Figure S 11 (a), the ELF map of $Li_{12}Al_3Si_4$ indicates that the excess electrons are also trapped in interstitial sites (see the red circle). The electronic structure of electride $Li_{12}Al_3Si_4$:5e$^-$ is shown in Fig. S 11 (b). Since the symmetry

operations are the same with C12A7:4e$^-$, $Li_{12}Al_3Si_4$:5e$^-$ also possesses the SDP and FDP on the high symmetry points H and P, respectively. However, unlike electride C12A7:4e$^-$, the FDP and FDP of electride $Li_{12}Al_3Si_4$:5e$^-$ are far away from the Femi level (above 0.6 eV). These fermions and their surface Fermi arc states are unlikely contribute to the conducting active in the system, as shown in Fig. S 11 (c). As a result, $Li_{12}Al_3Si_4$:5e$^-$ has a much higher ΔG_{H*} on the (001) surface than C12A7:4e$^-$ (1.90 eV *versus* 0.24 eV), as shown in Fig. S 11 (d).

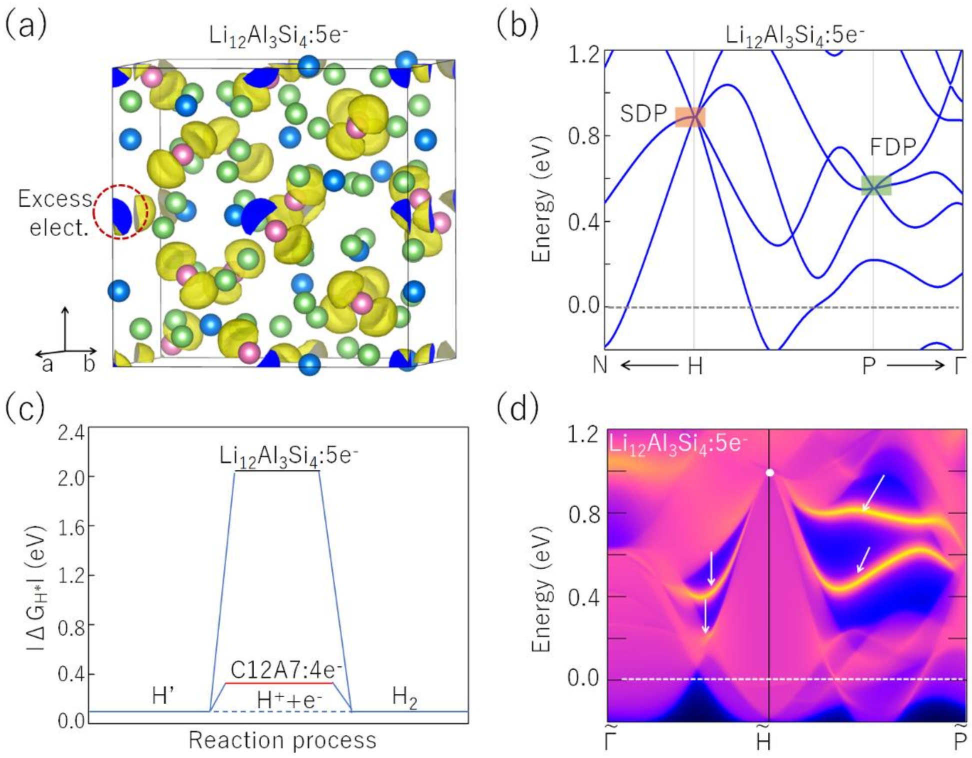

Figure S14 (a) The ELF map of electride $Li_{12}Al_3Si_4$:5e$^-$ with the isosurface values set as 0.8. (b) The electronic structure for electride $Li_{12}Al_3Si_4$:5e$^-$. (c) Volcano plot for IHP of electride $Li_{12}Al_3Si_4$:5e$^-$ in comparison with electride C12A7:4e$^-$. (d) The (001) surface states at specific paths for $Li_{12}Al_3Si_4$:5e$^-$. The Fermi arcs are pointed by the write arrows.

<u>12. Surface electronic density in C12A7:4e$^-$:</u>

As shown in Fig. S15 (a) and (b) below, the electronic density at the Fermi level for the (001) surface is found to be 2.7 times larger than that of the bulk.

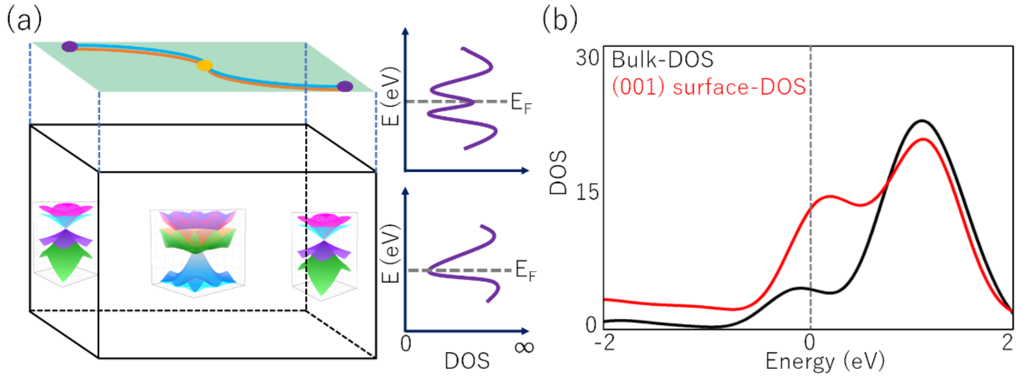

Figure S15 (a) multiple fermions in bulk (left lower panel) and the nearly flat non-trivial surface states on the surface (left upper panel) and their corresponding DOSs (right panels) and (b) comparison of the densities of states (DOSs) between the bulk phase and (001) surface of electride C12A7:4e$^-$.

13. The electronic band structure with spin-orbital coupling (SOC):

It is worth noting that due to the weak spin orbit coupling of electrides, the electronic band under SOC is placed in the supporting information. Based on the DFT calculations and symmetry analysis, when the SOC is not ignored, the six-fold degenerate point (SDP) on the high-symmetry point H split into an eight-fold degenerate point (EDP) and a four-fold degenerate point (FDP), as shown in Fig. S 12 (a). Besides, the FDP on the high-symmetry point P split into two three-fold degenerate points (TDPs) and two-fold degenerate point [see Fig. S 12 (b)]. Figure S 12 (c) shows the 3D band dispersions of EDP and TDP, respectively. Figure S 12 (d) shows the positions of EDP and TDP in Brillouin zone (BZ).

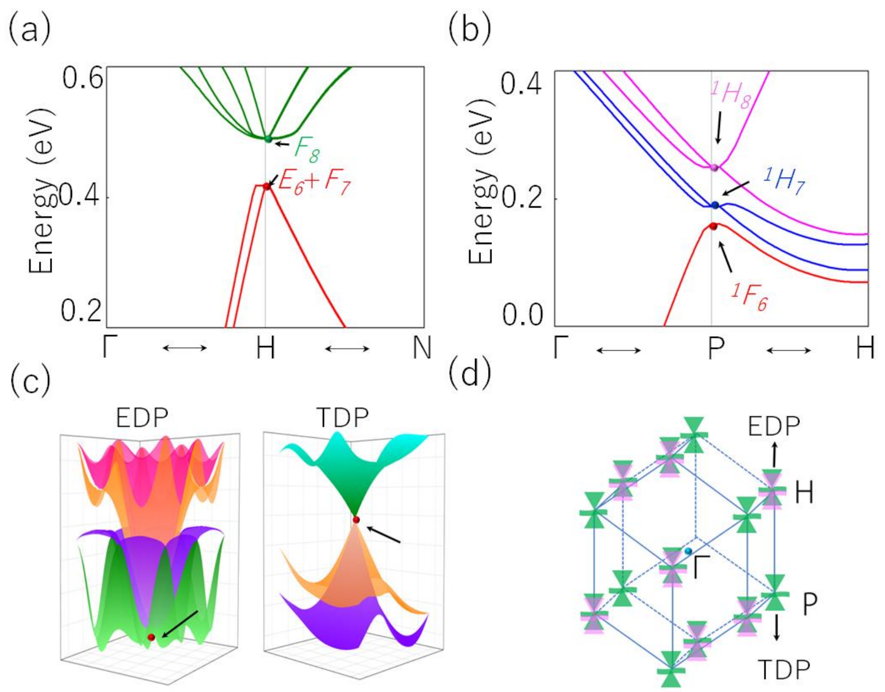

Figure S16 (a) and (b) show the locally enlarged band structure with considering SOC along high symmetry points H and P. In (a) and (b), the F_8, E_6, F_7, $^1H_8$, $^1H_7$, $^1F_6$ are irreducible representations of points H and P, respectively. (c) The 3D band dispersion bands of eight-fold and three-fold degenerate nodal points on points H and P, respectively. (d) The position of eight-fold and three-fold degenerate nodal points (EDP, TDP) in Brillouin zone (BZ).

Reference

[1] B. Bradlyn, L. Elcoro, J. Cano, M. G. Vergniory et al. Nature 547 298-305 (2017)

[2] S. Nie, B. A. Bernevig et al. Phys. Rev. Research. 3 L012028 (2021).

[3] T. R. Chang, S. Y. Xu, G. Chang, C. C. Lee et al. Nat. Commun. 7, 10639 (2016).

[4] A. Kanigel, M. R. Norman, M. Randeria et al. Nat. Phys. 2, 447 (2006).

[5] A. Kanigel, U. Chatterjee, M. Randeria et al. Phys. Rev. Lett. 99, 157001 (2007).
